# Supplementary material for: Stress-driven potentiation of lateral hypothalamic synapses onto ventral tegmental area dopamine neurons causes increased consumption of palatable food
Source: Nat Commun. 2022 Nov 12;13:6898. doi: 10.1038/s41467-022-34625-7 (PMC9653441; doi:10.1038/s41467-022-34625-7)
Supplement: Supplementary file 1 — Supplementary Information [file 41467_2022_34625_MOESM1_ESM.pdf]

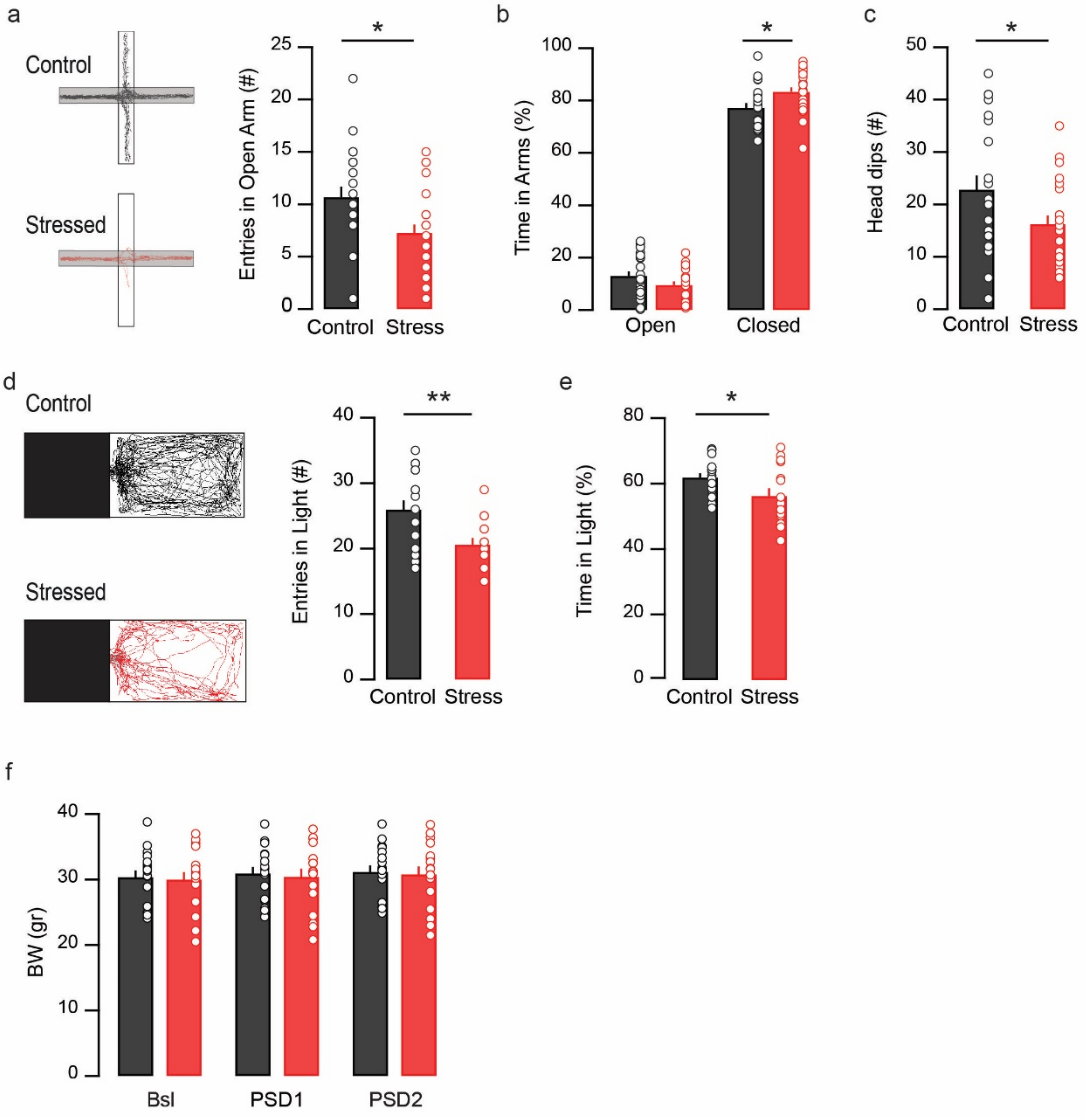

SUPPLEMENTARY FIGURE 1

Supplementary Figure 1. Social stress enhances anxiety levels in mice. (a) Number of entries in open arms decreased post stress in elevated plus maze (EPM). Left: representative movement trace of control (black) and stress (red) animals in EPM. Right: Averages +SEMs. (Control n=22 mice, Stress n=23 mice, One-Way ANOVA, $F_{(1,43)}=7.10$, $p=0.011$). (b) Time in open and closed arms as a percentage of total time in Elevated Plus Maze (EPM) showed a more anxious phenotype in stressed animals. Averages +SEMs (n control=22 mice, n stress=23 mice; One-Way ANOVA closed arms, $F_{(1,43)}=6.17$, $p=0.017$; One-Way ANOVA open arms $F_{(1,43)}=2.52$, $p=0.12$). (c) Number of head dips out of the open arms in the EPM decreased post stress. Averages +SEMs (n control=22 mice, n stress=23 mice, One-Way ANOVA $F_{(1,43)}=4.48$, $p=0.04$). (d) Number of entries in the light compartment decreased after two days of social stress in the Light Dark (LD)-box. Left: representative movement traces of control (black) and stress (red) animals in LD-box. Right: Averages +SEMs (n control=17 mice, n stress=15 mice, One-Way ANOVA $F_{(1,30)}=8.76$, $p=0.006$). (e) Averages +SEMs of time in light in LD-box as percentage of total time decreased post stress (n control=17 mice, n stress=15 mice, One-Way ANOVA $F_{(1,30)}=4.75$, $p=0.04$). (f) Bodyweight was not different between control and stressed animals subjected to a High fat high sugar diet. Averages +SEMs (n group=17 mice, RM Two-Way ANOVA. Main effect stress, $F_{(1,32)}=0.06$, $p=0.81$). All statistical tests were performed two-sided. *$p<0.05$, **$p<0.01$. Source data are provided as a Source Data file.

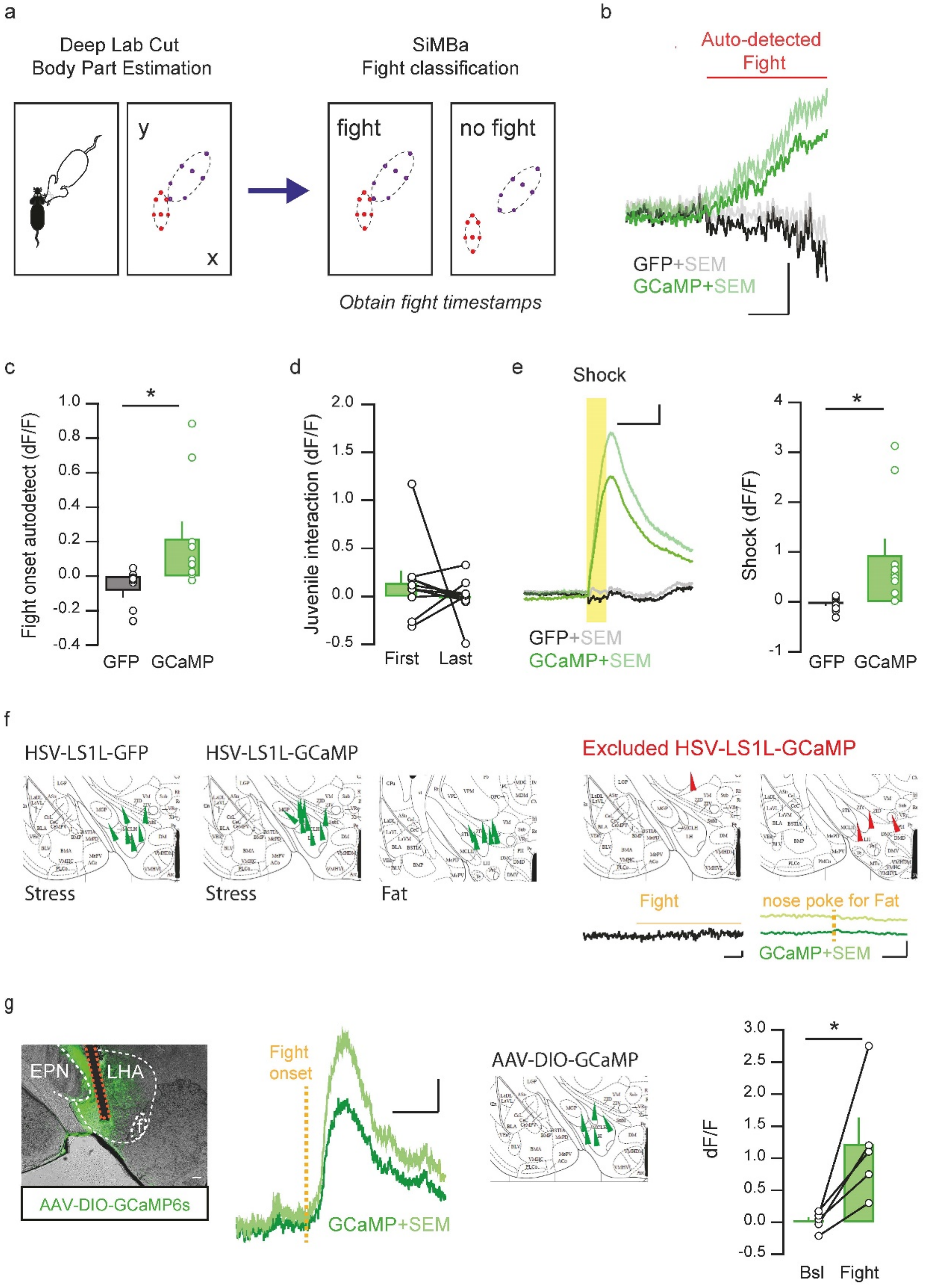

SUPPLEMENTARY FIGURE 2

<u>Supplementary Figure 2.</u> Stress sensitivity of LHA glutamatergic neurons projecting to VTA. (a) Schematic of supervised machine learning approach to obtain predictive classifiers of fight behavior. (b) Average recorded emitted fluorescent signal of GCaMP6s plus SEM (dF/F) (green) and control GFP (black) of LHA_{glu}-VTA neurons time-locked to fight onset of automatically detected fights (red line). Scale bars: 1 s, 0.25 dF/F. (c) Automatically-detected fights coincided with increased GCaMP6s signal compared to control (mice per group: GFP n=9, GCaMP n=10; One-Way ANOVA $F_{(1,17)}$=7.42, p=0.014). (d) Quantified peak signals of GCaMP of LHA_{glu}-VTA neurons during first and last juvenile with SEM (mice per group n=10, One-Way ANOVA $F_{(1,9)}$=0.58, ns). (e) Average recorded emitted fluorescent signal plus SEM of GCaMP6s (dF/F) (green; n=10) and control GFP (black; n=10) of LHA_{glu} neurons projecting to VTA time-locked to 1 s foot shock (yellow bar). Scale bars: 2 s, 0.25 dF/F. Right: Averages + SEMs of peak signals compared to baseline during shock of GCaMP and GFP control groups (mice per group n=10, One-Way ANOVA, $F_{(1,18)}$=7.01, p=0.016). (f) Left: Maps of optic fiber placements for LHA_{glu}-VTA GCaMP fiber photometric recordings during stress and nose poke for fat. Triangles indicate end of optic fiber (bottom) and angle of placement. Right: Histological maps of excluded animals due to misplaced fibers or lack of viral expression. Bottom: recorded traces during fight or fat intake of animals excluded (fight: n animals =1, fat n animals =3; average +SEM; orange line indicates onset of fight or nose poke for fat). Scale bars: 1 s, 0.2 dF/F. (g) LHA_{glu} neurons are activated during fight. Left: Viral expression of GCaMP6s in LHA_{glu} neurons (in green), with an optic fiber in the LHA. Anatomical reference points: f=fornix, EPN= Entopeduncular Nucleus, mt=mammillothalamic tract (Scale bar: 100 µm). Middle left: Average recorded emitted fluorescent signal of GCaMP6s plus SEM (dF/F) (mice n=5) of LHA_{glu}, time-locked to fights (orange dashed line). Scale bars: 1 s, 0.25 dF/F. Middle right: Map of optic fiber placements for LHA_{glu} GCaMP fiber photometric recordings during stress. Right: Quantified signals of GCaMP during baseline and fight (n mice =5, One-Way RM ANOVA, $F_{(1,4)}$=10.05, p=0.034). All statistical tests were performed two-sided. *p<0.05. Source data are provided as a Source Data file.

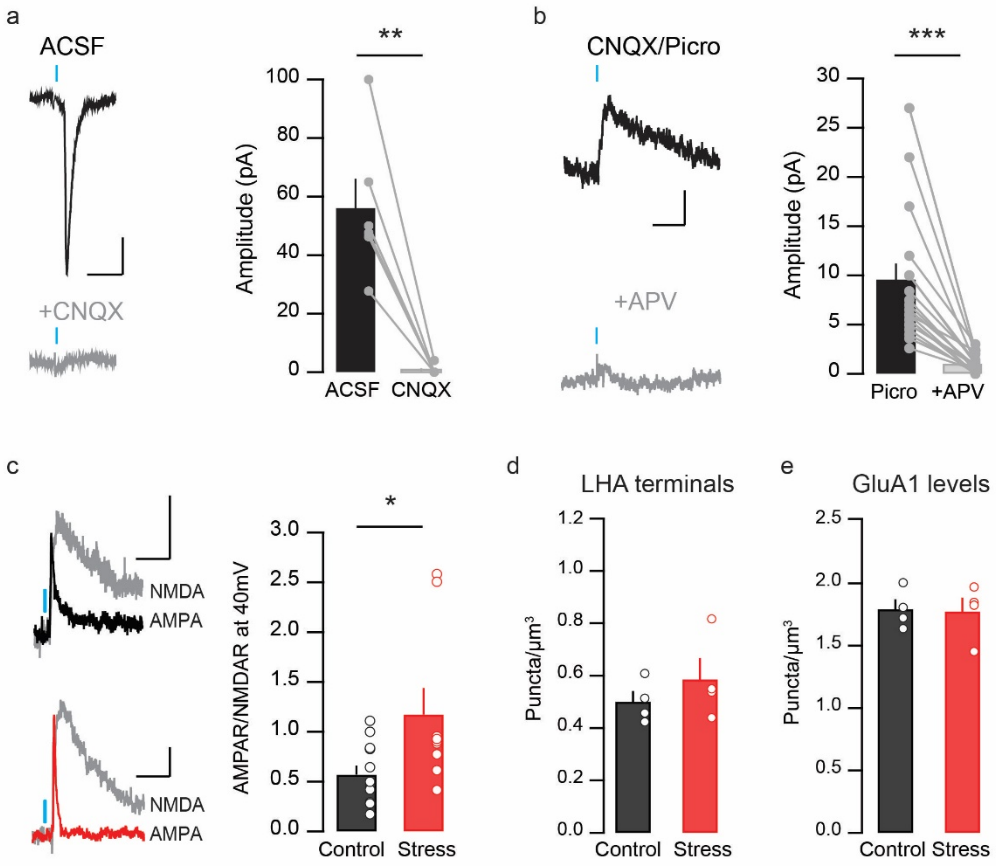

SUPPLEMENTARY FIGURE 3

__Supplementary Figure 3.__ **Validation of stress driven potentiation LHA_{glu}-VTA_{DA} synapses.** (a) LHA-VTA_{DA} opto-currents measured at -65 mV are blocked by AMPA/Kainate receptor antagonist CNQX. Left: Example traces of AMPAR current at -65 mV with and without CNQX. Scale bars: 10 pA, 20 ms. Right: Averages + SEMs (n cells=6, RM ANOVA, $F_{(1,5)}$=34.39, p=0.002). (b) LHA-VTA_{DA} opto-currents measured at +40 mV at 100 ms in the presence of $GABA_AR$ antagonist picrotoxin and AMPA/Kainate receptor antagonist CNQX is blocked by NMDAR antagonist APV. Left: example trace at +40 mV without (top) and with (bottom) APV. Scale bar: 20 pA, 50 ms. Right: Averages + SEMs (n cells=21, RM ANOVA, $F_{(1,20)}$=33.04, p=0.00001). (c) LHA_{glu}-VTA_{DA} AMPAR/NMDAR ratio (peak to peak) both at +40 mV in the presence of $GABA_AR$ antagonist Picrotoxin, with further pharmacological blockade of AMPAR/Kainate (CNQX) to isolate individual AMPAR (black or red) and NMDAR (gray) components. Left: example traces. Scale bars 40 pA, 50 ms. Right: Averages + SEMs (control n=11, stress n=9, One-Way ANOVA, $F_{(1,18)}$=5.18, p=0.035). (d) The density of LHA fibers in the VTA does not differ between mice subjected to control or stress conditions (averages +SEMs, mice per group n=4, One-Way ANOVA, $F_{(1,6)}$=0.22, p=0.66). (e) The density of overall (synaptic and non-synaptic combined) GluA1-AMPAR subunits in the VTA does not differ after stress (averages + SEMs, mice per group n=4, One-Way ANOVA, $F_{(1,6)}$=0.02, p=0.89). All statistical tests were performed two-sided. *p<0.05, **p<0.01, ***p<0.001. Source data are provided as a Source Data file.

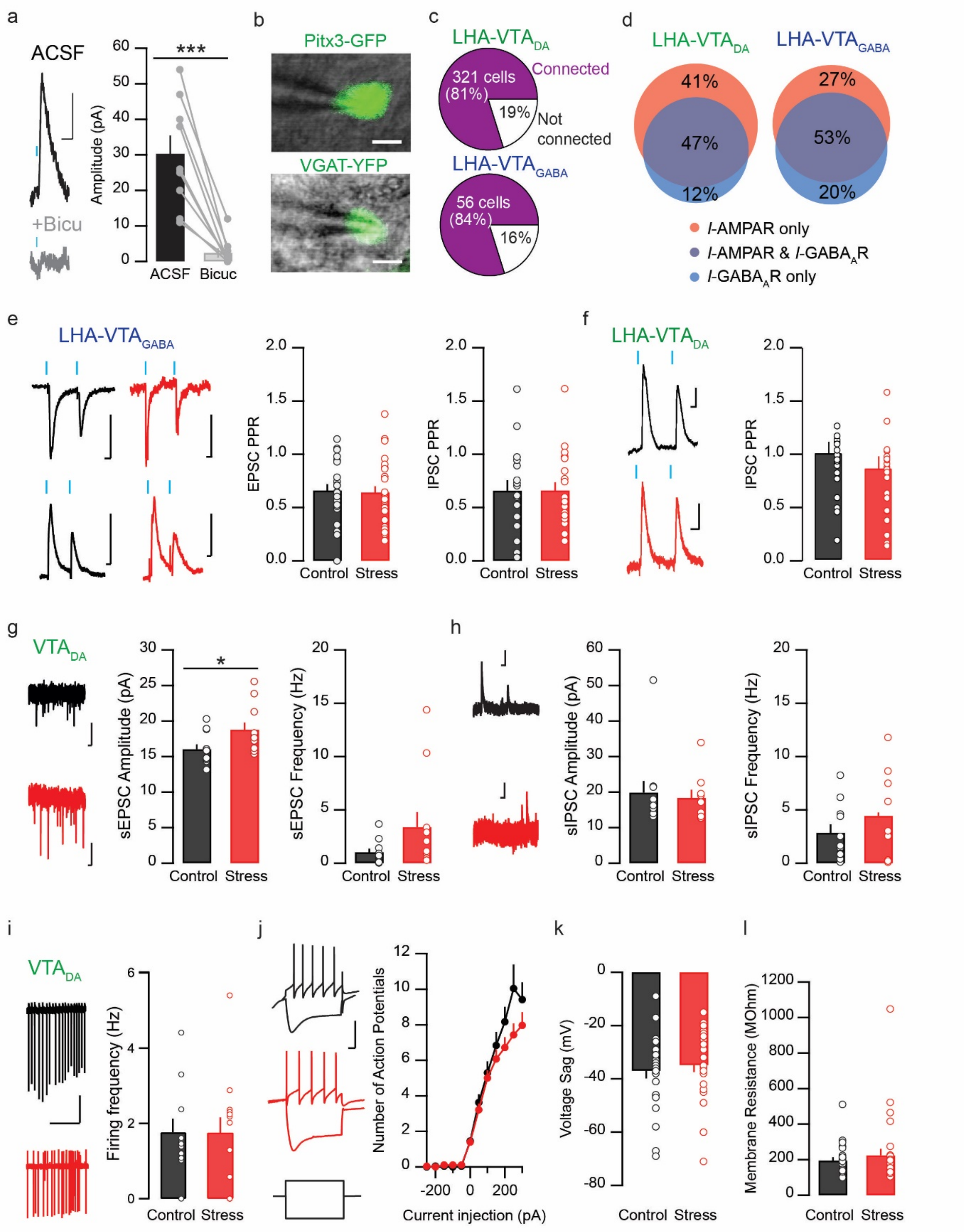

SUPPLEMENTARY FIGURE 4

<u>Supplementary Figure 4.</u> Stress plasticity is specific for $\mathbf{LHA_{glu}}$-$\mathbf{VTA_{DA}}$ synapses. (a) Opto-evoked currents at LHA-VTA_{DA} synapses when holding VTA_{DA} neurons at 0 mV are completely blocked by a $GABA_AR$ antagonist Bicuculline. Left: Example traces. Scale bars: 40 pA, 40 ms. Right: Averages + SEMs (n cells=9, RM ANOVA, $F_{(1,8)}$=39.21, p=0.0002). (b) Representative images of patched VTA_{DA} (top) and VTA_{GABA} (bottom) neurons with patch clamp recording electrode. Scale bars: 10 µm. (c) Connectivity of glutamatergic LHA input to VTA_{DA} neurons and VTA_{GABA} neurons. (d) Connectivity overview of LHA input types to VTA_{DA} neurons (n=49 total) and VTA_{GABA} neurons (n=64 total). (e) Paired pulse ratios (PPR) of LHA-VTA_{GABA} synapses. Left: example traces of PPR at either type of synapse. Scale bars: 25 pA, 20 ms. Middle: PPR at LHA_{glu}-VTA_{GABA} synapses does not differ due to stress (averages +SEMs, cells per group control n=23, stress n=26, One-Way ANOVA, $F_{(1,47)}$=0.05, p=0.82). Right: PPR of LHA_{GABA}-VTA_{GABA} neurons does not differ due to stress (control cells n=17, stress cells n=20, One-Way ANOVA, $F_{(1,35)}$=0.0006, p=0.89). (f) PPR of LHA_{GABA}-VTA_{DA} synapses does not change after stress. Left: example traces of PPR. Scale bars: 25 pA, 20 ms. Right: Averages + SEMs (control cells n=20, stress cells n=22, One-Way ANOVA, $F_{(1,40)}$=1.82, p=0.37). (g) Amplitude of sEPSCs onto VTA_{DA} neurons is increased after stress. Left: Example traces of sEPSCs. Scale bars: 20 pA, 100 ms. Right: Averages +SEMs for amplitude (cells per group n=11, One-Way ANOVA, $F_{(1,20)}$=5.08, p=0.036) and frequency (One-Way ANOVA, $F_{(1,20)}$=2.7, p=0.12). (h) sIPSCs onto VTA_{DA} neurons are not affected by stress. Left: Example traces of sIPSCs. Scale bars: 25 pA, 100 ms. Right: Averages +SEMs for amplitude (control cells n=11, stress cells n=9, One-Way ANOVA, $F_{(1,18)}$=0.12, p=0.73) and frequency (One-Way ANOVA $F_{(1,18)}$=1.06, p=0.32). (i) Spontaneous firing frequency of VTA_{DA} neurons recorded in cell-attached mode was not different due to stress. Left: example traces, Scale bars: 100 pA, 10s. Right Averages +SEMs (control cells n=12, stress cells n=13, One-Way ANOVA, $F_{(1,23)}$=0.07, p=0.80). (j) The number of action potentials of VTA_{DA} neurons as a function of injected current steps in current clamp did not differ between control and stress. Left: example traces (for injected current -200 pA and 150 pA). Scale bars: 50 mV, 100 ms. Right: Averages +SEMs (control cells n=24, stress cells n=28, RM Two-Way ANOVA. Interaction Stress x Current, $F_{(1.72,85.9)}$=2.18, p=0.13). (k) The voltage sag of VTA_{DA} neurons in response to -200 pA of injected current did not differ between stress and control. Averages +SEMs (control cells n=24, stress cells n=28, One-Way ANOVA, $F_{(1,50)}$=0.30, p=0.59). (l) The membrane resistance of VTA_{DA} neurons was not different for control versus stress. Averages +SEMs (control cells n=24, stress cells n=28, One-Way ANOVA, $F_{(1,50)}$=0.48, p=0.49). All statistical tests were performed two-sided. *p<0.05, ***p<0.001. Source data are provided as a Source Data file.

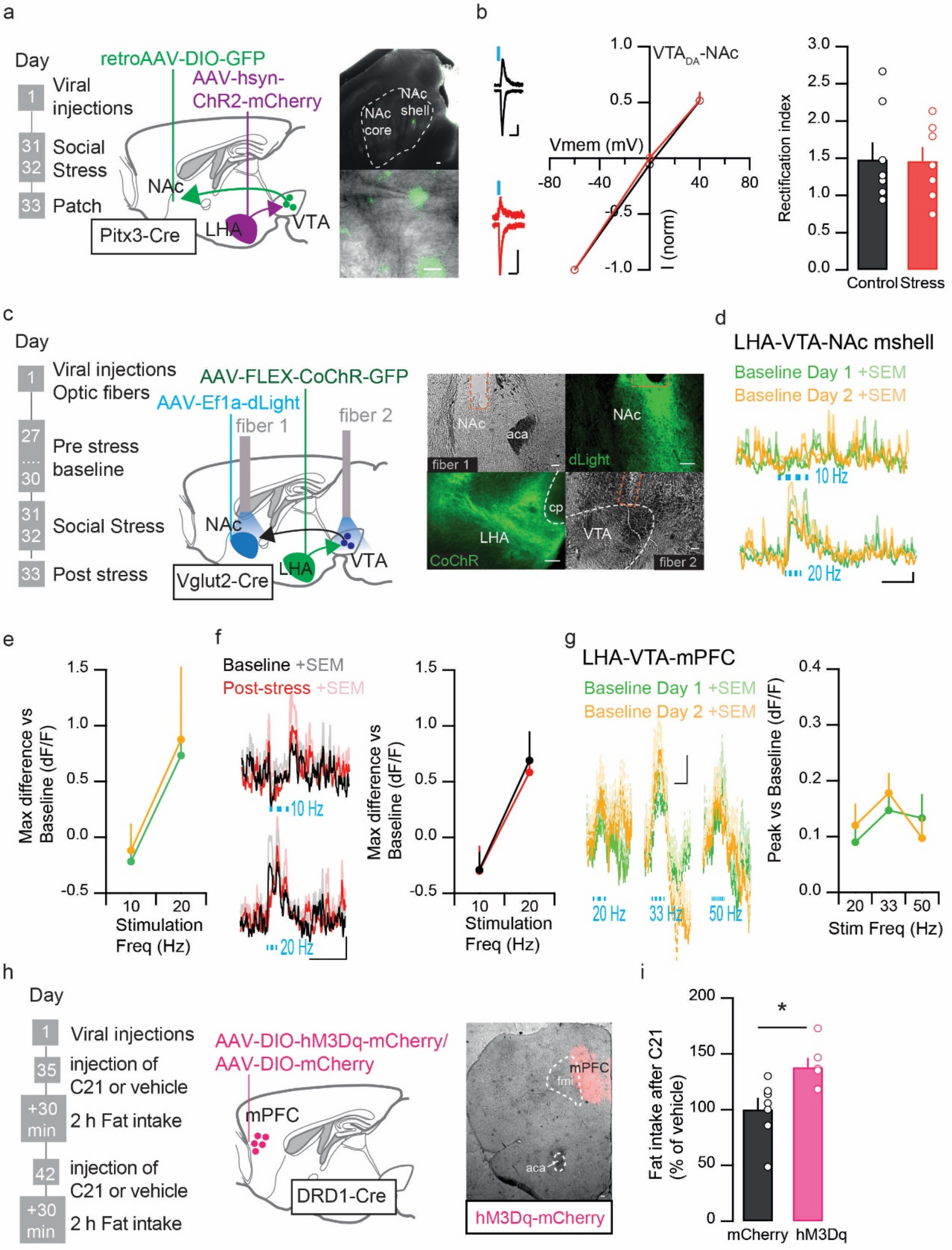

SUPPLEMENTARY FIGURE 5

<u>Supplementary Figure 5.</u> Stress does not potentiate LHA_{glu}-VTA_{DA}-NAc mshell synapses. (a) Schematic of patch clamp experiment of VTA_{DA} neurons projecting to NAc medial shell. Right: viral expression of retroAAV-DIO-GFP (+ diluted green beads for injection verification) in horizontal sections. Top: NAc scale bar: 100 µm. Bottom: VTA scale bar: 20 µm. (b) AMPAR Rectification index at LHA_{glu}-VTA_{DA}-NAc_{mshell} synapses does not changes as a function of stress. Left: example traces, scale bars: 50 ms, 10 pA. Middle: AMPAR current at -60 mV, 0 mV and +40 mV normalized to AMPAR current at -60 mV (n cells control=8, n cells stress=7). Right: Averages +SEMs (n cells control=8, n cells stress=7, KS-test, $D_{(1,14)}=0.43$, p=0.97). (c) Experimental timeline and schematic of *in vivo* LHA_{glu}-VTA driven dopamine release pre and post stress. Right: Representative example of virus expression and fiber placement. Scale bars: 100 µm. Anatomical reference points: aca=anterior commissure, anterior part, cp=cerebral peduncle. (d): Averages of LHA_{glu}-VTA driven emitted fluorescence of dLight in the NAc medial shell in response to 10 and 20 Hz stimulation on baseline day 1 (green) and baseline day 2 (orange) plus SEMs. Scale bars: 5 s, 0.2 dF/F. (e) Maximum difference emitted fluorescence opposed to baseline is stable over two baseline days (averages +SEMs, n=3 animals, RM Two-Way ANOVA, Main day effect. $F_{(1,2)}=2.62$, p=0.25). (f) Left: same as d for pre stress (black) and post stress (red). Right: Maximum difference emitted fluorescence (compared to baseline) is not altered by stress (n=5 animals, RM Two-way ANOVA. Main effect stress $F_{(1,4)}=0.34$, p=0.59). (g) Left: Averages +SEMs of LHA_{glu}-VTA driven emitted fluorescence of dLight in the mPFC in response to stimulation trains of 20, 33 and 50 Hz were stable over baseline days. Scale bar: 0.05 dF/F, 10 s. Right: Peak difference emitted fluorescence vs baseline (n=6 animals, RM Two-Way ANOVA. Main effect Day $F_{(1,5)}=0.029$; p=0.87). (h) Left: Timeline and schematic for fat intake with chemogenetic stimulation of $mPFC_{DRD1}$ neurons. Right: Viral expression of rAAV5-hSyn-DIO-hM3D(Gq)-mCherry in $mPFC_{DRD1}$ neurons. Scale bar: 100 µm. Anatomical reference points: fmi=forceps minor of the corpus callosum. (i) Stimulation of $mPFC_{Drd1}$ neurons enhances fat intake (averages +SEMs, n animals mCherry=7, n animals hM3Dq=6, one-way ANOVA. $F_{(1,12)}=8.0$, p=0.016). All statistical tests were performed two-sided. *p<0.05. Source data are provided as a Source Data file.

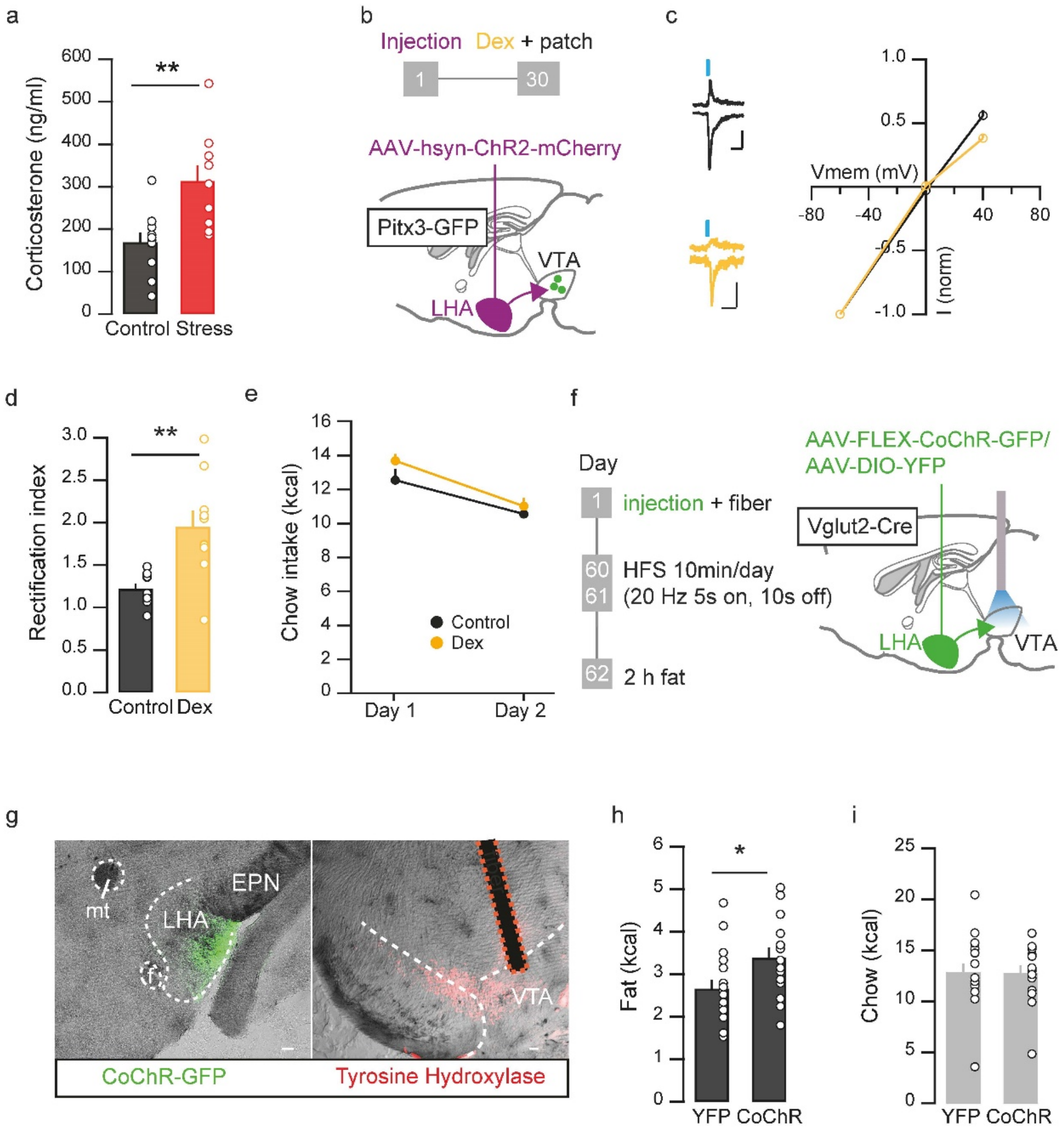

SUPPLEMENTARY FIGURE 6

<u>Supplementary Figure 6.</u> Mimicking LHA_{glu}-VTA stress plasticity enhances fat but not chow intake. (a) Corticosterone concentration in blood plasma increases in mice subjected to 20 s of fighting compared to control animals cohoused with a novel C57B6J (averages +SEMs, n animals control=10, n animals stress=9, One-Way ANOVA, $F_{(1,17)}$=10.40, p=0.005). (b) Experimental timeline and schematic of brain slice incubation with glucocorticoid receptor (GR) agonist dexamethasone (Dex). (c) AMPAR Rectification index increases at LHA_{glu}-VTA_{DA} synapses after Dex incubation. Left: example traces, scale bars: 25 ms, 10 pA. Right: AMPAR current at -60 mV, 0 mV and +40 mV normalized to AMPAR current at -60 mV (n cells control=9, n cells Dex=10). (d) AMPAR Rectification index Averages + SEMs (n cells control=9, n cells Dex=10, KS-test, $D_{(1,17)}$=1.96, p=0.001). (e) Chow intake does not change by *in vivo* Dex infusion (averages +SEMs, n animals control=6, n animals Dex= 5, RM Two-way ANOVA. Main effect of Dex, $F_{(1,9)}$=3.49, p=0.31). (f) Experimental timeline and schematic of *in vivo* High Frequency Stimulation (HFS: 20 Hz, 5 s on, 10 s off) effects on fat intake. (g) Representative images of viral expression and fiber placement. Scale bars: 100 µm. Anatomical reference points; f=fornix, mt=mammillothalamic tract, EPN= entopeduncular Nucleus. (h) Two days of *in vivo* optogenetic HFS of LHA_{glu}-VTA enhances fat intake (averages +SEMs, n animals GFP=17, n animals CoChR=15, One-way ANOVA. $F_{(1,30)}$=5.25, p=0.029). (i) Two days of HFS of LHA_{glu}-VTA does not alter chow intake (averages +SEMs, n animals GFP=17, n animals CoChR=15, One-way ANOVA. $F_{(1,30)}$=0.007, p=0.93). All statistical tests were performed two-sided. *p<0.05. Source data are provided as a Source Data file.

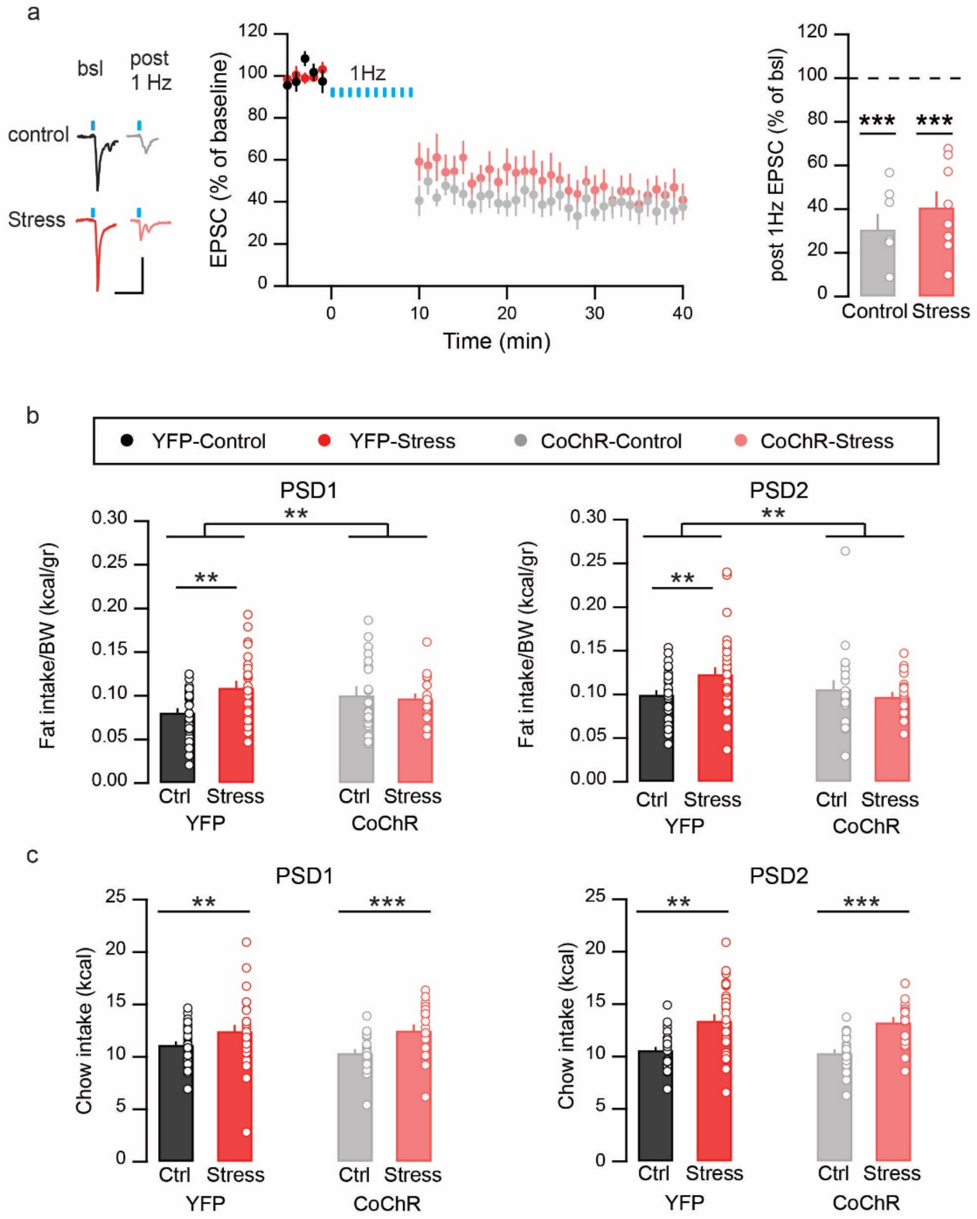

SUPPLEMENTARY FIGURE 7

__Supplementary Figure 7.__ **Weakening LHA_{glu}-VTA synaptic transmission prevents stress-driven fat intake but does not affect chow intake.** (a) Opto-stimulated LHA_{glu}-VTA_{DA} EPSCs decrease due to a 1 Hz 10 minute stimulation protocol. Bar graph: Averaged EPSCs last 10 min post stim. Left: example traces, scale bars: 25 pA, 25 ms. Right: Averages +SEMs (n cells control=7, RM ANOVA, $F_{(1,6)}$=95.44, p=0.00007, n cells stress= 8, RM ANOVA, $F_{(1,7)}$=64.21, p=0.00009). (b) Total fat intake per BW (kcal/gr) on PSD1 and PSD2 in 2 h fat access in mice that were either stressed or not, and with or without a prior *in vivo* LFS (averages +SEMs, n animals control YFP=30, stress YFP=30, control CochR=21, stress CoChR=19; RM Two-way ANOVA. Interaction stress x Virus Type, $F_{(1,96)}$=7.66, p=0.007; YFP-Control vs YFP-Stress $F_{(1,58)}$=12.85, p=0.001; CoChR-Control vs CoChR-Stress $F_{(1,38)}$=0.44, p=0.51). (c) As (b) but for Chow intake (kcal) on PSD1 and PSD2 in 22 h chow access (averages +SEMs, n animals control YFP=30, stress YFP=30, control CochR=21, stress CoChR=19; RM Two-Way ANOVA. Interaction Stress x Virus type $F_{(1,96)}$=0.27, p=0.61. Main stress effect $F_{(1,96)}$=25.42, p=0.000002; YFP-Control vs YFP-Stress $F_{(1,58)}$=10.54, p=0.0002; CoChR-Control vs CoChR-Stress $F_{(1,38)}$=19.16, p=0.00009). All statistical tests were performed two-sided. **p<0.01, ***p<0.001. Source data are provided as a Source Data file.

SUPPLEMENTARY TABLE 1

Figure	Variable	Group	N mice	N data points	Statistical test	Comparison	DF	Test value	P value
1b	Fat intake 24h (Kcal)	Control	17	17		Day x Group interaction	1,32	F=0.005	p=0.95
		Stress	17	17	Repeated Measures Two-Way ANOVA	Day Main effect	1,32	F=0.80	p=0.80
						Group Main effect	1,32	F=18.44	p=0.0002
					Post-Hoc contrasts	PSD1: Control vs Stress	1,32	F=11.19	p=0.002
						PSD2: Control vs Stress	1,32	F=10.40	p=0.003
1c	Sugar intake 24h (Kcal)	Control	17	17		Day x Group interaction	1,32	F=0.1	p=0.75
		Stress	17	17	Repeated Measures Two-Way ANOVA	Day Main effect	1,32	F=10.55	p=0.003
						Group Main effect	1,32	F=8.951	p=0.005
					Post-Hoc contrasts	PSD1: Control vs Stress	1,32	F=6.39	p=0.017
						PSD2: Control vs Stress	1,32	F=9.54	p=0.004
1d	Chow intake 24h (Kcal)	Control	17	17		Day x Group interaction	1,32	F=7.409	p=0.0104
		Stress	17	17	Repeated Measures Two-Way ANOVA	Day Main effect	1,32	F=1.08	p=0.3
						Group Main effect	1,32	F=2.238	p=0.14
					Post-Hoc contrasts	PSD1: Control vs Stress	1,32	F=0.84	p=0.37
						PSD2: Control vs Stress	1,32	F=10.34	p=0.003
1e	Total intake 24h (Kcal)	Control	17	17		Day x Group interaction	1,32	F=3.9	p=0.06
		Stress	17	17	Repeated Measures Two-Way ANOVA	Day Main effect	1,32	F=0.284	p=0.60
						Group Main effect	1,32	F=13.76	p=0.0008
					Post-Hoc contrasts	PSD1: Control vs Stress	1,32	F=15.64	p=0.0004
						PSD2: Control vs Stress	1,32	F=0.52	p=0.47
1f	Palatable intake 24h (% of total)	Control	17	17		Day x Group interaction	1,32	F=5.4	p=0.03
		Stress	17	17	Repeated Measures Two-Way ANOVA	Day Main effect	1,32	F=1.5	p=0.23
						Group Main effect	1,32	F=15.4	p=0.0004
					Post-Hoc contrasts	PSD1: Control vs Stress	1,32	F=2.08	p=0.16
						PSD2: Control vs Stress	1,32	F=23.26	p=0.00003
1g	Fat intake 2h (Kcal)	Control	10	10		Day x Group interaction	1,18	F=0.37	p=0.55
		Stress	10	10	Repeated Measures Two-Way ANOVA	Day Main effect	1,18	F=3.003	p=0.10
						Group Main effect	1,18	F=7.071	p=0.016
					Post-Hoc contrasts	PSD1: Control vs Stress	1,18	F=2.80	p=0.11
						PSD2: Control vs Stress	1,18	F=7.33	p=0.014
1h	Chow intake 22h (Kcal)	Control	10	10		Day x Group interaction	1,18	F=1.94	p=0.18
		Stress	10	10	Repeated Measures Two-Way ANOVA	Day Main effect	1,18	F=8.29	p=0.01
						Group Main effect	1,18	F=3.15	p=0.0928
					Post-Hoc contrasts	PSD1: Control vs Stress	1,18	F=3.4	p=0.08
						PSD2: Control vs Stress	1,18	F=1	p=0.3
1i	Total intake 24h (Kcal)	Control	10	10		Day x Group interaction	1,18	F=0.97	p=0.09
		Stress	10	10	Repeated Measures Two-Way ANOVA	Day Main effect	1,18	F=3.2	p=0.34
						Group Main effect	1,18	F=12.18	p=0.0026
					Post-Hoc contrasts	PSD1: Control vs Stress	1,18	F=7.75	p=0.012
						PSD2: Control vs Stress	1,18	F=7.84	p=0.011

Figure	Variable	Group	N mice	N data points	Statistical test	Comparison	DF	Test value	P value
2c	Fight onset (dF/F)	GFP	10	10	One-Way ANOVA	GFP vs GCaMP	1,18	F=12.03	p=0.003
		GCaMP	10	10					
2d	Fight offset (dF/F)	GFP	10	10	One-Way ANOVA	GFP vs GCaMP	1,18	F=3.65	p=0.072
		GCaMP	10	10					
2e	High velocity bout (dF/F)	GFP	9	9	One-Way ANOVA	GFP vs GCaMP	1,16	F=1.00	p=0.33
		GCaMP	9	9					
2f	Juvenile interaction (dF/F)	GFP	10	10	One-Way ANOVA	GFP vs GCaMP	1,18	F=1.75	p=0.20
		GCaMP	10	10					
2h	peak dF/F FP LHAglu-VTA	control pre vs post	4	4		Time * Stress	1,10	F=0.535	p=0.482
		stress pre vs post	8	8	RM ANOVA	Time	1,10	F=0.004	p=0.951
						stress	1,10	F=0.338	p=0.54

Figure	Variable	Group	N mice	N data points	Statistical test	Comparison	DF	Test value	P value
3c	AMPAR/NMDAR	Control	4	15	One-Way ANOVA	Control vs Stress	1,23	F=14.72	p=0.001
		Stress	6	10					
3d	AMPAR rectification index	Control	10	18	One-Way ANOVA	Control vs Stress	1,33	F=5.81	p=0.02
		Stress	8	17					
3e	EPSCP PPR	Control	15	31	One-Way ANOVA	Control vs Stress	1,60	F=0.02	p=0.89
		Stress	12	31					
3h	puncta per um3	Control	4	4	One-Way ANOVA	Control vs Stress	1,6	F=18.26	p=0.005
		Stress	4	4					
3i	puncta per um3	Control	4	4	One-Way ANOVA	Control vs Stress	1,6	F=14.9	p=0.008
		Stress	4	4					

Figure	Variable	Group	N mice	N data points	Statistical test	Comparison	DF	Test value	P value
4b	VTA DA neurons: GABAaR / AMPAR	Control	14	23	Kolmogorov-Smirnov Test	Control vs Stress	1,46	D=0.499	p=0.003
		Stress	13	25					
4c	VTA GABA neurons: GABAaR / AMPAR	Control	9	25	Kolmogorov-Smirnov Test	Control vs Stress	1,52	D=0.34	p=0.074
		Stress	9	29					
4e	VTA DA-PFC neurons: AMPAR rectification index	Control	3	7	Kolmogorov-Smirnov Test	Control vs Stress	1,15	D=1.701	p=0.006
		Stress	4	9					
4h	Peak dF/F of 20 Hz stimulation	Control				Frequency x Group interaction	2,10	F=1.49	p=0.28
		Stress	6	6	Repeated Measures Two-Way ANOVA	Frequency Main effect	2,10	F=2.62	p=0.12
						Group Main effect	1,5	F=6.92	p=0.047
						20 Hz: bsl vs Stress	1,5	F=6.50	p=0.051
					Post-Hoc contrasts	33 Hz: bsl vs Stress	1,5	F=3.36	p=0.12
						50 Hz: bsl vs Stress	1,5	F=4.4	p=0.09

Figure	Variable	Group	N mice	N data points	Statistical test	Comparison	DF	Test value	P value
5a	AMPAR amplitude	Vehicle	4	27	Kolmogorov Smirnov Test	Vehicle vs Dexamethasone	1,52	D=0.37	p=0.036
		Dexamethasone	5	27					
5c	Fat intake 2h (Kcal)	Vehicle	6	6		Day x Group interaction	1,9	F=0.004	p=0.95
		Dexamethasone	5	5	Repeated Measures Two-Way ANOVA	Day Main effect	1,9	F=1.11	p=0.32
						Group Main effect	1,9	F=7.03	p=0.026
					Post-Hoc contrasts	PSD1: Veh vs Dex	2,18	t=1.77	p=0.18
						PSD2: Veh vs Dex	2,18	t=1.86	p=0.16
5f	AMPAR amplitude	Control	9	26		stress vs stress +1Hz	1,42	D=0.61	p=0.0002
		Stress	6	22					
		Stress + 1 Hz	7	24	Kolmogorov Smirnov Test				
5g	Fat intake	YFP-Control	30	30		stress group x opsin interaction	1,96	F=7.16	p=0.009
		YFP-Stress	30	30		stress main	1,96	F=3.4	p=0.07
		CoChR-Control	21	21		opsin main	1,96	F=0.05	p=0.83
		CoChR-Stress	19	19	Repeated Measures Two-Way ANOVA	day main effect	1,96	F=4.94	p=0.03
						Day x stress	1,96	F=0.30	p=0.59
						Day x opsin	1,96	F=2.32	p=0.13
						day x stress x opsin	1,96	F=0.00	p=0.99
					posthoc contrast				
						day	1,58	F=9.69	p=0.003
					RM ANOVA YFP	day x stress	1,58	F=0.19	p=0.66
						stress	1,58	F=12.78	p=0.001
						day	1,38	F=0.18	p=0.68
					RM ANOVA CochR	day x stress	1,38	F=0.12	p=0.74
						stress	1,38	F=0.28	p=0.60

Figure	Variable	Group	N mice	N data points	Statistical test	Comparison	DF	Test value	P value
S Fig 1a	Entries open arm (#)	Control	22	22		Control vs Stress	1,43	F=7.10	p=0.011
		Stress	23	23	One way ANOVA				
S Fig 1b	Time in arms (% of total time)	Control	22	22		ct vs stress stress open arm	1,43	F=2.52	p=0.12
		Stress	23	23	One way ANOVA				
						ct vs stress closed arm	1,43	F=6.17	p=0.017
					One way ANOVA				

	Variable	Group	N mice	N data points	Statistical test	Comparison	DF	Test value	P value
S Fig 1c	Head dips (#)	Control<br>Stress	22<br>23	22<br>23	One way ANOVA	Control vs Stress	1,43	F=4.48	p=0.04
S Fig 1d	Entries Light (#)	Control<br>Stress	17<br>15	17<br>15	One way ANOVA	Control vs Stress	1,30	F=8.76	p=0.006
S Fig 1e	Time in light (% of total time)	Control<br>Stress	17<br>15	17<br>15	One way ANOVA	Control vs Stress	1,30	F=4.75	p=0.037
S Fig 1f	Bodyweight (gr)	Control<br>Stress	17<br>17	17<br>17	Repeated Measures Two-Way ANOVA	Day x Group interaction<br>Day Main effect<br>Group Main effect	1,32<br>1,32<br>1,32	F=0.21<br>F=10.02<br>F=0.06	p=0.81<br>p=0.0001<br>p=0.81

	Variable	Group	N mice	N data points	Statistical test	Comparison	DF	Test value	P value
S Fig 2c	Fight onset autodetected (dF/F)	GFP<br>GCaMP	9<br>10	9<br>10	One-Way ANOVA	GFP vs GCaMP	1,17	F=7.42	p=0.014
S Fig 2d	peak dF/F first vs last juvenile interaction	GCaMP LHAglu-VTA	10	10	One-way RM ANOVA	1st vs last juvenile interaction	1,9	F=0.58	p=0.46
S Fig 2e	Shock onset (dF/F)	GFP<br>GCaMP	10<br>10	10<br>10	One-Way ANOVA	GFP vs GCaMP	1,18	F=7.01	p=0.016
S Fig 2g	dF/F LHAglu fight	GCaMP LHAglu	5	5	One-way RM ANOVA	base vs fight	1,4	F=10.05	p=0.034

	Variable	Group	N mice	N data points	Statistical test	Comparison	DF	Test value	P value
S Fig 3a	AMPA verification (pA)	pre CNQX<br>post CNQX	3	6	Repeated Measures ANOVA	pre vs post CNQX	1,5	F=34.39	p=0.002
S Fig 3b	NMDA verification (pA)	pre APV<br>post APV	11	21	Repeated Measures ANOVA	pre vs post APV	1,20	F=33.05	p=0.00013
S Fig 3c	AMPAR/NMDAR at 40mV	Control<br>Stress	4<br>6	11<br>9	One-Way ANOVA	Control vs Stress	1,18	F=5.18	p=0.035
S Fig 3d	LHA Punta/ɥm^{3}	Control<br>Stress	4<br>4	4<br>4	One-Way ANOVA	Control vs Stress	1,6	F=0.22	p=0.66
S Fig 3e	GluA1 Punta/ɥm^{3}	Control<br>Stress	4<br>4	4<br>4	One-Way ANOVA	Control vs Stress	1,6	F=0.02	p=0.89

	Variable	Group	N mice	N data points	Statistical test	Comparison	DF	Test value	P value
S Fig 4a	GABA A verification (pA)	pre bicuc<br>post bicuc	5	9	Repeated Measures ANOVA	pre vs post bicuc	1,8	F=39.21	p=0.0002
S Fig 4c	PPR at LHA glu-VTA GABA	Control<br>Stress	8<br>7	23<br>26	One-way ANOVA	Control vs Stress	1,47	F=0.05	p=0.82
	PPR at LHA GABA-VTA GABA	Control<br>Stress	8<br>6	17<br>20	One-way ANOVA	Control vs Stress	1,35	F=0.0006	p=0.98
S Fig 4d	PPR at LHA GABA-VTA DA	Control<br>Stress	10<br>11	20<br>22	One-Way ANOVA	Control vs Stress	1,40	F=1.82	p=0.37
S Fig 4e	sEPSC amplitude VTA DA (pA)	Control<br>Stress	5<br>6	11<br>11	One-Way ANOVA	Control vs Stress	1,20	F=5.08	p=0.036
	sEPSC Frequency VTA DA (Hz)	Control<br>Stress	5<br>6	11<br>11	One-Way ANOVA	Control vs Stress	1,20	F=2.71	p=0.12
S Fig 4f	sIPSC amplitude VTA DA (pA)	Control<br>Stress	5<br>6	11<br>9	One-Way ANOVA	Control vs Stress	1,18	F=0.12	p=0.73
	sIPSC Frequency VTA DA (Hz)	Control<br>Stress	5<br>6	11<br>9	One-Way ANOVA	Control vs Stress	1,18	F=1.06	p=0.32
S Fig 4g	firing frequency VTA DA (Hz)	Control<br>Stress	5<br>5	12<br>13	One-Way ANOVA	Control vs Stress	1,23	F=0.07	p=0.80
S Fig 4h	Nr action potentials VTA DA (#)	Control<br>Stress	5<br>7	24<br>28	Repeated Measures Two-Way ANOVA	main Stress<br>stress x currentstep<br>current step	1,50<br>1.72, 85.9<br>11,50	F=1.43<br>F= 2.18<br>F=158	p=0.24<br>p=0.13<br>p<0.000001
S Fig 4i	Voltage sag VTA DA (mV)	Control<br>Stress	5<br>7	24<br>28	One-Way ANOVA	Control vs Stress	1,50	F=0.30	p=0.59
S Fig 4j	membrane resistance VTA DA (Mohm)	Control<br>Stress	5<br>7	24<br>28	One-Way ANOVA	Control vs Stress	1,50	F=0.48	p=0.49

	Variable	Group	N mice	N data points	Statistical test	Comparison	DF	Test value	P value
S Fig 5b	VTA DA-Nac neurons: AMPAR rectification index	Control<br>Stress	3<br>4	8<br>7	Kolmogorov-Smirnov Test	Control vs Stress	1,14	D=0.43	p=0.97
S Fig 5e	Max difference opposed to baseline (dF/F)	baseline day 1<br>baseline day 2	3<br>3	3<br>3	Repeated Measures Two-Way ANOVA	Day<br>Freq<br>day x freq	1,2<br>1,2<br>1,2	F=2.62<br>F=1.52<br>F=0.035	p=0.25<br>p=0.34<br>p=0.89
S Fig 5f	Max difference opposed to baseline (dF/F)	pre stress<br>post stress	5<br>5	5<br>5	Repeated Measures Two-Way ANOVA	stress<br>Freq<br>stress x freq	1,4<br>1,4<br>1,4	F=0.34<br>F=5.82<br>F=3.22	p=0.59<br>p=0.072<br>p=0.15
S Fig 5g	Peak opposed to baseline (dF/F)	baseline day 1<br>baseline day 2	6<br>6	6<br>6	Repeated Measures Two-Way ANOVA	Day<br>Freq<br>day x freq	1,5<br>1,5<br>1,5	F=0.029<br>F=1.77<br>F=0.76	p=0.87<br>p=0.22<br>p=0.49
S Fig 5i	fat intake (% c21 day/ vehicle) PFC drd1	mCherry<br>Gq-dreadd	7<br>6	7<br>6	one way ANOVA	mCherry vs CoChR	1,12	F=8.008	p=0.016

	Variable	Group	N mice	N data points	Statistical test	Comparison	DF	Test value	P value
S Fig 6a	corticosterone (ng/ml)	control<br>stress	10<br>9	10<br>9	One-Way ANOVA	Control vs Stress	1,17	F=10.40	p=0.005
S Fig 6d	VTA DA neurons: Dex induced AMPAR RI	Control<br>Dexamethasone	2<br>2	9<br>10	Kolmogorov-Smirnov Test	Control vs Stress	1,17	D=1.959	p=0.001
S Fig 6e	Chow intake (kcal)	vehicle<br>dexamethasone	6<br>5	6<br>5	Repeated Measures Two-Way ANOVA	Dex<br>day<br>Dex x Day	1,9<br>1,9<br>1,9	F=3.49<br>F=50.89<br>F=1.10	p=0.31<br>p<0.0001<br>p=0.32
S Fig 6h	post HFS LHAglu-VTA Fat intake	YFP<br>CoChR	17<br>15	17<br>15	one way ANOVA	YFP vs CoChR	1,30	F=5.25	p=0.029
S Fig 6i	post HFS LHAglu-VTA chow intake	YFP<br>CoChR	17<br>15	17<br>15	one way ANOVA	YFP vs CoChR	1,30	F=0.007	p=0.93

	Variable	Group	N mice	N data points	Statistical test	Comparison	DF	Test value	P value
S Fig 7a	post 1 Hz EPSC	control pre 1 hz<br>control post 1hz	7	7	Repeated Measures ANOVA	pre vs post 1Hz	1,6	F=95.44	p=0.00007
	post 1 Hz EPSC	stress pre 1 hz<br>stress post 1hz	6	8	Repeated Measures ANOVA	pre vs post 1Hz	1,7	F=64.21	p=0.00009
S Fig. 7b	Fat intake/BW (kcal/gr)	YFP-Control<br>YFP-Stress<br>CoChR-Control<br>CoChR-Stress	30<br>30<br>21<br>19	30<br>30<br>21<br>19	Repeated Measures Two-Way ANOVA	stress group x opsin interaction<br>stress main<br>opsin main<br>day main effect<br>Day x stress<br>Day x opsin<br>day x stress x opsin	1,96<br>1,96<br>1,96<br>1,96<br>1,96<br>1,96<br>1,96	F=7.66<br>F=2.96<br>F=0.26<br>F=4.72<br>F=0.31<br>F=2.42<br>F=0.00	p=0.007<br>p=0.09<br>p=0.62<br>p=0.03<br>p=0.58<br>p=0.12<br>p=0.98
					post hoc contrast				
					RM ANOVA YFP	day<br>day x stress<br>stress	1,58<br>1,58<br>1,58	F=9.44<br>F=0.19<br>F=12.85	p=0.003<br>p=0.66<br>p=0.001
					RM ANOVA CochR	day<br>day x stress<br>stress	1,38<br>1,38<br>1,38	F=0.14<br>F=0.12<br>F=0.44	p=0.71<br>p=0.73<br>p=0.51
S Fig.7c	Chow intake (kcal)	YFP-Control<br>YFP-Stress<br>CoChR-Control<br>CoChR-Stress	30<br>30<br>21<br>19	30<br>30<br>21<br>19	RM ANOVA	stress group x opsin interaction<br>stress main<br>opsin main<br>day main effect<br>Day x stress<br>Day x opsin<br>day x stress x opsin	1,96<br>1,96<br>1,96<br>1,96<br>1,96<br>1,96<br>1,96	F=0.27<br>F=25.42<br>F=0.42<br>F=1.57<br>F=6.59<br>F=0.10<br>F=0.59	p=0.61<br>p=0.000002<br>p=0.52<br>p=0.21<br>p=0.012<br>p=0.75<br>p=0.45
					post hoc contrast				
					RM ANOVA YFP	day<br>day x stress<br>stress	1,58<br>1,58<br>1,58	F=0.52<br>F=6.72<br>F=10.54	p=0.47<br>p=0.012<br>p=0.002
					RM ANOVA CochR	day<br>day x stress<br>stress	1,38<br>1,38<br>1,38	F=1.09<br>F=1.43<br>F=19.16	p=0.30<br>p=0.24<br>p=0.00009
