# Supplementary material for: Stress-driven potentiation of lateral hypothalamic synapses onto ventral tegmental area dopamine neurons causes increased consumption of palatable food
Source: Nat Commun. 2022 Nov 12;13:6898. doi: 10.1038/s41467-022-34625-7 (PMC9653441; doi:10.1038/s41467-022-34625-7)
Supplement: Supplementary file 3 — Reporting Summary [file 41467_2022_34625_MOESM3_ESM.pdf]

nature portfolio

Corresponding author(s): F.J. Meye

Last updated by author(s): Oct 24, 2022

Reporting Summary

Nature Portfolio wishes to improve the reproducibility of the work that we publish. This form provides structure for consistency and transparency in reporting. For further information on Nature Portfolio policies, see our Editorial Policies and the Editorial Policy Checklist.

Statistics

For all statistical analyses, confirm that the following items are present in the figure legend, table legend, main text, or Methods section.

n/a	Confirmed	
☐	☒	The exact sample size (*n*) for each experimental group/condition, given as a discrete number and unit of measurement
☐	☒	A statement on whether measurements were taken from distinct samples or whether the same sample was measured repeatedly
☐	☒	The statistical test(s) used AND whether they are one- or two-sided<br>*Only common tests should be described solely by name; describe more complex techniques in the Methods section.*
☒	☐	A description of all covariates tested
☐	☒	A description of any assumptions or corrections, such as tests of normality and adjustment for multiple comparisons
☐	☒	A full description of the statistical parameters including central tendency (e.g. means) or other basic estimates (e.g. regression coefficient) AND variation (e.g. standard deviation) or associated estimates of uncertainty (e.g. confidence intervals)
☐	☒	For null hypothesis testing, the test statistic (e.g. *F*, *t*, *r*) with confidence intervals, effect sizes, degrees of freedom and *P* value noted<br>*Give P values as exact values whenever suitable.*
☒	☐	For Bayesian analysis, information on the choice of priors and Markov chain Monte Carlo settings
☐	☒	For hierarchical and complex designs, identification of the appropriate level for tests and full reporting of outcomes
☒	☐	Estimates of effect sizes (e.g. Cohen's *d*, Pearson's *r*), indicating how they were calculated

Our web collection on statistics for biologists contains articles on many of the points above.

Software and code

Policy information about availability of computer code

Data collection	Fiber photometry data were collected using a Doric set up. The data acquisition unit and fiber photometry console were connected to the dual channel programmable LED driver (400mA/V in analogue mode), which in turn controlled the maximum power (950 mA) output to the light sources: a blue LED for excitation of calcium dependent GCaMP6s (440-490 nm). Individual LED power was set at 120-160 µW in the brain. The emitted fluorescence, went through the same 400 µm patch cable back to the minicube, where it was split by two dichromatic mirrors (420- 450 nm and 460-490 nm), and was sent via 600 µm core cables to photoreceivers. Photon-to-electron conversion took place in AC low mode (gain 2x1010 V/A, Bandwidth 30-750 Hz) and signal was sent back to the fiber photometry console for demodulation. Recordings took place in lock-in mode, using 208 Hz as reference frequencies for blue (465 nm) light channel. Data were acquired at 12 kilosamples/s and decimated 50 times using Doric Neuroscience Studio.<br><br>For voltage or current clamp recordings, signal was amplified, low-pass filtered at 2.9 kHz with a 4-pole Bessel filter, and digitized at 20 kHz with an EPC10 dual patch-clamp USB amplifier (HEKA Elektronik GmbH). Data were acquired using PatchMaster v2x90.2software<br><br>Behavioral data collection for both EPM and LD box was done with Ethovision video tracking (version 9; Noldus). Automatic fight scoring was done with an automated pipeline, using DeepLabCut (version 2.0.6) for pose estimation, and SiMBA for machine learning (behavioral) classification.
Data analysis	Data were analyzed with Noldus (Ethovision V9.0), SPSS (IBM V26), Python v3.8.8, Igor Pro-8 (Wavemetrics, USA), MedPC-4 (Med Associates Inc) and Mini Analysis v6.0 (Synaptosoft, USA).

For manuscripts utilizing custom algorithms or software that are central to the research but not yet described in published literature, software must be made available to editors and reviewers. We strongly encourage code deposition in a community repository (e.g. GitHub). See the Nature Portfolio guidelines for submitting code & software for further information.

Data

Policy information about availability of data

All manuscripts must include a data availability statement. This statement should provide the following information, where applicable:

- Accession codes, unique identifiers, or web links for publicly available datasets
- A description of any restrictions on data availability
- For clinical datasets or third party data, please ensure that the statement adheres to our policy

The data collected during the study are available from the corresponding author upon request.

Human research participants

Policy information about studies involving human research participants and Sex and Gender in Research.

Reporting on sex and gender	N/A
Population characteristics	N/A
Recruitment	N/A
Ethics oversight	N/A

Note that full information on the approval of the study protocol must also be provided in the manuscript.

Field-specific reporting

Please select the one below that is the best fit for your research. If you are not sure, read the appropriate sections before making your selection.

☒ Life sciences ☐ Behavioural & social sciences ☐ Ecological, evolutionary & environmental sciences

For a reference copy of the document with all sections, see nature.com/documents/nr-reporting-summary-flat.pdf

Life sciences study design

All studies must disclose on these points even when the disclosure is negative.

Sample size	Sample size was predetermined on the basis of published studies, experimental pilots and in-house expertise.<br>PMID: 25643299, PMID: 27348214, PMID: 33867112.
Data exclusions	For automated fight detection with DLC and SimBA, one animal in the GFP group was excluded as it did not reach the criteria for minimum detected fight duration and two animals were excluded from the velocity analysis as they did not reach the high speed criterion.<br>For fiber photometry measurements 4 animals were exlcuded because of either misplacement of fiber optic or failure of calcium indicator expression in the correct brain region.
Replication	All experiments were performed at least twice, leading to the same result.
Randomization	Animals were randomly assigned to treatment groups before testing.
Blinding	Investigators were blinded to group allocation during data acquisition and analysis.

Reporting for specific materials, systems and methods

We require information from authors about some types of materials, experimental systems and methods used in many studies. Here, indicate whether each material, system or method listed is relevant to your study. If you are not sure if a list item applies to your research, read the appropriate section before selecting a response.

Materials & experimental systems

n/a	Involved in the study
☐	☒ Antibodies
☒	☐ Eukaryotic cell lines
☒	☐ Palaeontology and archaeology
☐	☒ Animals and other organisms
☒	☐ Clinical data
☒	☐ Dual use research of concern

Methods

n/a	Involved in the study
☒	☐ ChIP-seq
☒	☐ Flow cytometry
☒	☐ MRI-based neuroimaging

Antibodies

Antibodies used	anti-Vglut2 (guinea pig, 1:1000; Millipore, Germany; AB2251),<br>anti-synapsin1 (clone D12G5) (rabbit, 1:400; Cell Signaling Technology, USA; #5297).<br>anti-GluA1 (clone RH95) (mouse, 1:200; Millipore, Germany; MAB2263).<br>anti-tyrosine hydroxylase (clone LNC1) (mouse, 1:400/ 1:2000; Millipore, Germany; MAB318, #3782107).<br>anti-GFP (chicken;1:1000 or 1:2000, GFP-1020, Aves, #GFP87948)<br>anti-RFP (rabbit, 1:500, Rockland, 600-401-379, #46317)
Validation	anti-Vglut2 (guinea pig, 1:1000; Millipore, Germany; AB2251),<br>anti-synapsin1 (clone D12G5) (rabbit, 1:400; Cell Signaling Technology, USA; #5297).<br>anti-GluA1 (clone RH95) (mouse, 1:200; Millipore, Germany; MAB2263).<br>anti-tyrosine hydroxylase (clone LNC1) (mouse, 1:400/ 1:2000; Millipore, Germany; MAB318, #3782107).<br>anti-GFP (chicken;1:1000 or 1:2000, GFP-1020, Aves, #GFP87948)<br>anti-RFP (rabbit, 1:500, Rockland, 600-401-379, #46317)<br>were validated in: PMID: 24019494, PMID: 30279456, PMID: 27348214, PMID: 21764912, PMID: 30560948

Animals and other research organisms

Policy information about studies involving animals; ARRIVE guidelines recommended for reporting animal research, and Sex and Gender in Research

Laboratory animals	In all experiments naïve adult male mice were used (20-35 g, >6 weeks). C57Bl6J (Jax #664), DR1D-Cre (Jax #28298), Pitx3-GFP mice were a kind gift from Meng Li (MRC Clinical Science Center)54. Pitx3-Cre mice were a kind gift from Marten Smidt (University of Amsterdam)55. Vglut2-Cre (Jax #28863) and VGAT-Cre (Jax #016962) animals were bred in house but originated from the Jackson Laboratory. The proven-breeder Swiss-CD1 mice (35-45 g, >12 weeks) were purchased from Janvier (France).
Wild animals	The study did not involve wild animals.
Reporting on sex	Since social stress was the main experimental manipulation, and this occurs naturally towards male but not female mice, we focused on male mice in this study.
Field-collected samples	The study did not involve field collected samples.
Ethics oversight	Experiments were approved by the Animal Ethics Committee of Utrecht University and the Dutch Central Authority for Scientific Procedures on Animals (CCD), and were conducted in agreement with the Dutch law (Wet op de Dierproeven, 2014) and the European regulations (Guideline 86/609/EEC).

Note that full information on the approval of the study protocol must also be provided in the manuscript.
